# Disparities in socioeconomic status and neighborhood characteristics affect all-cause mortality in patients with newly diagnosed hypertension in Korea: a nationwide cohort study, 2002–2013

**DOI:** 10.1186/s12939-015-0288-2

**Published:** 2016-01-08

**Authors:** Kyoung Hee Cho, Sang Gyu Lee, Chung Mo Nam, Eun Jung Lee, Suk-Yong Jang, Seon-Heui Lee, Eun-Cheol Park

**Affiliations:** Department of Public Health, Graduate School, Yonsei University, Seoul, Korea; Institute of Health Services Research, College of Medicine, Yonsei University, Seoul, Korea; Graduate School of Public Health, Yonsei University, Seoul, Korea; Department of Biostatistics, College of Medicine, Yonsei University, Seoul, Korea; Graduate School of Social Welfare, Yonsei University, Seoul, Korea; Department of Preventive Medicine, College of Medicine, Yonsei University, Seoul, Korea; Department of Nursing Science, College of Nursing, Gachon University, Seongnam, Korea; Department of Preventive Medicine and Institute of Health Services Research, Yonsei University College of Medicine, 50 Yonsei-ro, Seodaemun-gu, Seoul, 120-752 Korea

**Keywords:** Socioeconomic status, Neighborhood deprivation, All-cause mortality, Hypertension

## Abstract

**Background:**

Previous studies have shown that contextual factors and individual socioeconomic status (SES) were associated with mortality in Western developed countries. In Korea, there are few empirical studies that have evaluated the association between SES and health outcomes.

**Methods:**

We conducted cohort study to investigate the socioeconomic disparity in all-cause mortality for patients newly diagnosed with hypertension in the setting of universal health care coverage. We used stratified random sample of Korean National Health Insurance enrollees (2002–2013). We included patients newly diagnosed with hypertension (*n* = 28,306) from 2003–2006, who received oral medication to control their hypertension. We generated a frailty model using Cox’s proportional hazard regression to assess risk factors for mortality.

**Results:**

A total of 7,825 (27.6%) of the 28,306 eligible subjects died during the study period. Compared to high income patients from advantaged neighborhoods, the adjusted hazard ratio (HR) for high income patients from disadvantaged neighborhoods was 1.10 (95% CI, 1.00–1.20; *p*-value = 0.05). The adjusted HR for middle income patients who lived in advantaged versus disadvantaged neighborhoods was 1.17 (95% CI, 1.08–1.26) and 1.27 (95% CI, 1.17–1.38), respectively. For low income patients, the adjusted HR for patients who lived in disadvantaged neighborhoods was higher than those who lived in advantaged neighborhoods (HR, 1.35; 95% CI, 1.22–1.49 vs HR, 1.28; 95% CI, 1.16–1.41).

**Conclusions:**

Neighborhood deprivation can exacerbate the influence of individual SES on all-cause mortality among patients with newly diagnosed hypertension.

**Electronic supplementary material:**

The online version of this article (doi:10.1186/s12939-015-0288-2) contains supplementary material, which is available to authorized users.

## Background

Hypertension is a chronic disease considered to be a major public health challenge [1–3] and is a key risk factor in the development of stroke, myocardial infarction, heart failure, and renal failure [4]. A socioeconomic gradient of risk factors for hypertension has been observed in a variety of settings. Previous studies that examined socioeconomic disparities related to the incidence or prevalence of hypertension have found that individuals with low income [5, 6], lower education [6–9], blue-collar occupation [6, 7], and living in disadvantaged neighborhoods [10, 11] face a higher probability of dying from complications of hypertension.

Most studies that explain the inverse relationship between socioeconomic status (SES) and risk factors for hypertension have come from the developed countries. The results of these studies demonstrated a relationship between individual socioeconomic characteristics and health status, where higher SES correlated with better health [12–15]. In comparison, other studies have reported that the characteristics of the neighborhoods in which the patients reside and the contextual factors independently affect individual health status. However, other studies have stated that neighborhood characteristics are the result of an aggregation of the relationships between the socioeconomic status and the health status of the individual [16]. An inverse relationship between mortality and neighborhood characteristics was found in Alameda County, 18 counties of Nova Scotia in Canada [17, 18]; in particular, low income individuals living in advantaged neighborhoods had higher mortality rates relative to low income individuals living in disadvantaged neighborhoods. The authors suggested “differential access to resources” as an explanation for these findings [18]. However, because Korea first implemented universal health care coverage in 1989 and has operated by a national tax system, there has been a greater redistribution of income in the population [19], resulting in smaller income inequalities than in the United States.

The aim of this study was to investigate the socioeconomic disparity at both individual and regional levels in all-cause mortality among patients with newly diagnosed hypertension using hierarchical modeling in a setting of universal health care coverage.

## Methods

### Data source for the study

This study used data from the Korean National Health Insurance (KNHI) claims database from 2002–2013 and the 2005 Korean Census. The National Health Insurance Corporation collects cohort data representative of Korea’s population. These data included the information on 1,025,340 subjects, and represented a stratified random sample selected according to age, sex, region, health insurance type, income quintiles, and individual total medical costs (based on the 2002 data). The database also included the information on reimbursement for each medical service, comprised of basic patient information, an identifier for the clinic or hospital, disease code, costs incurred, results of health screening, past/family health history, health behaviors, and information related to death. We focused especially on the characteristics of the neighborhoods in which patients resided. Ethical approval for this study was granted by the Institutional Review Board of the Graduate School of Public Health, Yonsei University.

### Selection of sample population

**Fig. 1:**
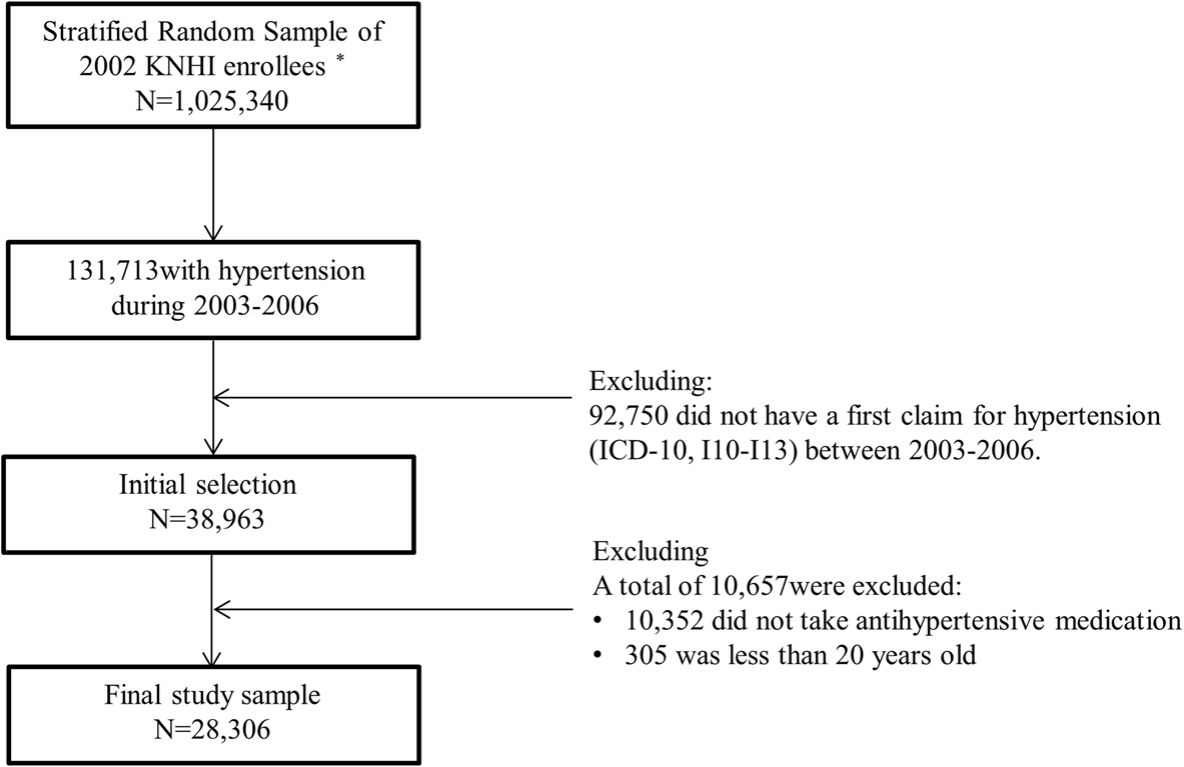
Flowchart for sample selection

We identified 131,713 individuals with hypertension between 2002 and 2013 in the KNHI enrollee database. Of these, 38,963 subjects with hypertension (I10-I13; International Classification of Disease, 10^th^ edition) newly diagnosed between 2003 and 2006 were selected. Hypertension patients consisted as following: primary hypertension (*n* = 24,809; I10), hypertensive heart disease (*n* = 2,791; I11), hypertensive renal disease (*n* = 297; I12), and hypertensive heart and renal disease (*n* = 409; I13). The proportion of patients with primary hypertension was approximately 90% (Additional file 1: Table S1). We confirmed that the diagnoses were new by verifying a lack of hypertension claims between 2002 and 2005, an initial hypertension claim between 2003 and 2006, and an absence of hypertension in the health history prior to the year of diagnosis. We included subject data collected over a minimum of 7 years and a maximum of 10 years. Of the 38,963 subjects initially selected, 10,657 were excluded: 305 patients were less than 20 years-old and 10,352 patients did not take antihypertensive medication. These exclusion criteria were necessary to determine the actual hypertension patients. The final study sample included 28,306 participants (Fig. 1).

### Dependent variable

The outcome variable was the survival time from the date of the diagnosis to the date of death or study end-date. We defined mortality as all-cause mortality, as identified from the death certificate data in the national death registry.

### Individual SES

We used the average monthly insurance premium as a proxy variable for household income. In Korea, the type of health insurance is classified as national health insurance or medical aid. Individuals qualify for medical aid if their household income is less than $600 per month based on a single household. If the household income is more than $600 per month, individuals qualify for national health insurance. Individuals who have national health insurance provided by their employer pay a monthly insurance premium according to annual salary, and those who are self-employed pay a premium according to property value. Individuals who qualified for the national health insurance were distributed between the 1st percentile and 100th percentile, and those who had medical aid were classified into the 0 percentile. We arbitrarily categorized individual household income into three groups (low, 0–20th percentile; middle, 21st–80th percentile; high, 81st–100th percentile). We divided into five groups as quintile according to household income. When we categorized high and low group using quintile, those belonging upper 20 percentile and lower 20 percentile were categorized to high and low, respectively.

### Neighborhood deprivation index

A summary measure was used to characterize the neighborhood-level deprivation. We used the modified Carstairs index [20] for measuring neighborhood deprivation using the census data from 2005. For the original calculation of the Carstairs index in previous studies, four variables from census data were used: 1) residents in households headed by unskilled workers, 2) unemployed males, 3) residents in overcrowded households, and 4) residents without a car. However, because we could not determine ‘residents without a car’ from the census data, according to Lee’s study’ methodology [21], we replaced ‘residents without car’ with ‘residents who rent their homes’. The neighborhood deprivation index was calculated at the Si (city), Gun (county) and Gu (borough) levels by merging the four basic indicators according to the method used for calculating the Carstairs index. Si, Gun, and Gu were geographical units we used to provide coverage across all smaller areas in Korea. We calculated a z-score at the Si, Gun, and Gu levels using the mean and standard deviation of the four indicators. A z-score was calculated by subtracting the mean from the observed value for each indicator, dividing it into the standard deviation, and then summing the four standardized z-scores. Disadvantaged and advantaged neighborhoods were distinguished on the basis of the median neighborhood deprivation index.

### Covariates

The covariates for our study were age (20–49, 50–59, 60–69, or ≥70 years), sex, residential area (metropolitan, urban or rural), Charlson Comorbidity Index (CCI) (0, 1, 2, or ≥3) [22], the number of risk factors (none, with diabetes or dyslipidemia, with diabetes and dyslipidemia), disability (normal, mild, severe), and the number of health screenings during the follow-up period (1, 2, 3 or 4). Only the comorbidity component of the CCI was calculated, and all diagnostic information was collected from inpatient and outpatient billing data at the time of diagnosis.

### Statistical analysis

Descriptive statistics were computed for all variables. The Chi-square test was used to calculate frequencies and percentages for categorical variables. The survival probability for all-cause mortality was estimated by the Kaplan-Meier product limit method, and the log-rank test was used to stratify SES. To investigate the association between individual-level and regional-level SES and all-cause mortality, we performed survival analysis using a Cox proportional hazards frailty model, which included random effects to deal with the covariates hierarchy denoted. This approach tests for a hospital effect as a random effect [23], which can be thought of as a “frailty”, increasing a region’s susceptibility to short survival time when it is large, and decreasing this susceptibility when it is small. We tested the variance and p-value for mortality among regions and determined that the variance was 0.016 and p-value was 0.003; therefore, we used the frailty model.

The equation λ (t|**x**) = *z*λ0(t)exp(**x**β) describes the frailty model where **x** represents the covariates matrix, β is the fixed effect vector, and Z is a random variable representing an unknown random effect related to regions, with the unit mean and variance ξ. These random effects act multiplicatively on the baseline hazard, and large values of ξ reflect a great degree of heterogeneity among regions. For model distribution purposes, we assumed that the frailties were distributed according to a gamma distribution. One attractive feature of the gamma distribution is that it is mathematically tractable [24]. The proportional hazards assumptions were tested using scaled Schoenfeld residuals and no violation was found. All of the statistical analyses were performed using SAS 9.3 software.

## Results

Of the 28,306 eligible subjects, 7,825 (27.6%) died during the study period and 20,481 (72.4%) survived (Table 1). There were significant differences between the two groups for all of the individual patient characteristics (age, sex, health insurance type, income, CCI, residential area, number of risk factors, disability, and the number of health screenings during study period).

**Table 1:** Baseline characteristics of patients with newly diagnosed hypertension

	Total	Alive		Dead		*P*-value
Characteristics	*N* = 28,306	*N* = 20,481	(72.4)	*N* = 7,825	(27.6)	
Age, N (%)						
20 ~ 49	5,769	5,434	(94.2)	335	(5.8)	<.0001
50 ~ 59	6,251	5,588	(89.4)	663	(10.6)	
60 ~ 69	7,977	6,131	(76.9)	1,846	(23.1)	
≥70	8,309	3,328	(40.0)	4,981	(60.0)	
Sex, N (%)						
Male	13,632	9,712	(71.2)	3,920	(28.8)	0.0001
Female	14,674	10,769	(73.4)	3,905	(26.6)	
Health insurance type, N (%)						
National health insurance	27,681	20,123	(72.7)	7,558	(27.3)	<.0001
Medical aid	625	358	(57.3)	267	(42.7)	
Income, N (%)						
Low (≤20th percentile)	4,801	3,192	(66.5)	1,609	(33.5)	<.0001
Middle (21st–80th percentile)	14,541	10,766	(74.0)	3,775	(26.0)	
High (≥81st percentile)	8,964	6,523	(72.8)	2,441	(27.2)	
Carstairs index, N (%)						
Disadvantaged neighborhood (below median)	15,855	11,458	(72.3)	4,397	(27.7)	0.6101
Advantaged neighborhood (above median)	12,451	9,023	(72.5)	3,428	(27.5)	
Combined individual household income level-neighborhood deprivation, N (%)						
High-Advantaged neighborhood	4,177	3,050	(73.0)	1,127	(27.0)	<.0001
High-Disadvantaged neighborhood	4,787	3,473	(72.5)	1,314	(27.5)	
Middle-Advantaged neighborhood	6,246	4,639	(74.3)	1,607	(25.7)	
Middle-Disadvantaged neighborhood	8,295	6,127	(73.9)	2,168	(26.1)	
Low-Advantaged neighborhood	2,028	1,334	(65.8)	694	(34.2)	
Low-Disadvantaged neighborhood	2,773	1,858	(67.0)	915	(33.0)	
Residential area, N (%)						
Metropolitan	12,470	9,303	(74.6)	3,167	(25.4)	<.0001
Urban	12,039	8,712	(72.4)	3,327	(27.6)	
Rural	3,797	2,466	(65.0)	1,331	(35.0)	
Charlson comorbidity index, N (%)						
0–1	15,875	12,842	(80.9)	3,033	(19.1)	<.0001
2	5,584	4,106	(73.5)	1,478	(26.5)	
3	3,121	1,992	(63.8)	1,129	(36.2)	
≥4	3,726	1,541	(41.4)	2,185	(58.6)	
Number of risk factors, N (%)						
None	11,600	7,344	(63.3)	4,256	(36.7)	
with diabetes or dyslipidemia	13,232	10,075	(76.1)	3,157	(23.9)	
with diabetes and dyslipidemia	3,474	3,062	(88.1)	412	(11.9)	
Disability, N (%)						
Normal	25,361	18,835	(74.3)	6,526	(25.7)	<.0001
Mild disability	1,950	1,260	(64.6)	690	(35.4)	
Severe disability	995	386	(38.8)	609	(61.2)	
Number with health screenings during follow-up period, N (%)						
1	16,022	9,338	(58.3)	6,684	(41.7)	<.0001
2	4,062	3,419	(84.2)	643	(15.8)	
≥3	8,222	7,724	(93.9)	498	(6.1)	

**Fig. 2:**
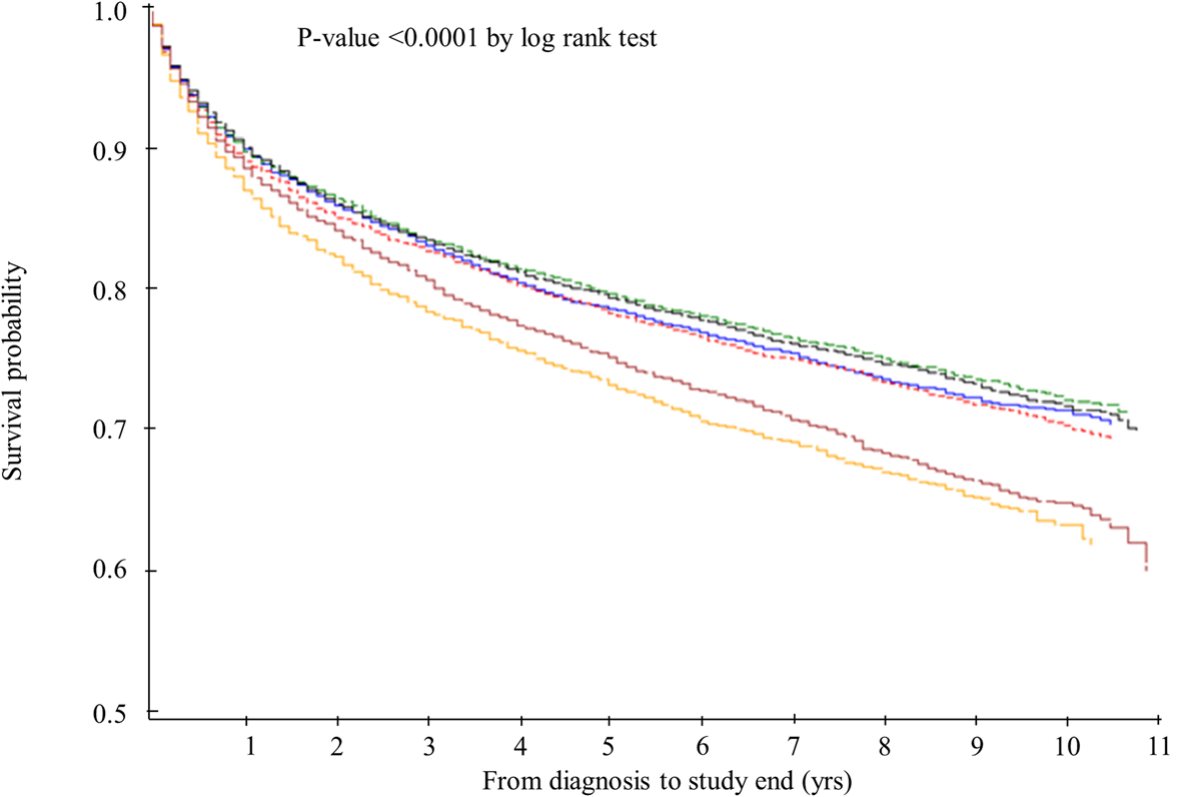
Survival probability for all-cause mortality, stratified to individual household income with advantaged and disadvantaged neighborhoods.  High within Advantaged neighborhoods-8.4 years*;  Middle within Advantaged neighborhoods-8.6 years*;  Low within Advantaged neighborhoods-7.7 years*;  High within Disadvantaged neighborhoods-8.4 years*;  Middle within Disadvantaged neighborhoods-8.7 years*;  Low within Disadvantaged neighborhoods-8.3 years*. *, Mean survival

By Kaplan-Meier analysis, the mean years of survival for low income individuals in disadvantaged neighborhoods was 8.3, while the mean years of survival for low income individuals in advantaged neighborhoods was 7.7 (p-value <0.0001 by log-rank test; Fig. 2).

Table 2 shows the results from the Cox regression analysis, which did not combine individual and neighborhood SES after controlling for all covariates. Compared to a high income reference group, the adjusted HR for low income and middle income groups was 1.25 (95% CI, 1.17–1.34) and 1.16 (95% CI, 1.10–1.22), respectively. In addition, the adjusted HR of patients in a disadvantaged neighborhood was 1.08 (95% CI, 1.02–1.15), compared with patients in an advantaged neighborhood.

**Table 2:** Hazard ratio for all-cause mortality among patients with newly diagnosed hypertension

	Unadjusted		Adjusted	
Characteristics	HR	95% CI	HR	95% CI
Age				
20 ~ 49	1.00		1.00	
50 ~ 59	1.88	(1.65–2.15)	2.35	(2.06–2.68)
60 ~ 69	4.41	(3.93–4.96)	4.85	(4.31–5.46)
≥70	15.49	(13.87–17.30)	12.60	(11.26–14.11)
Sex				
Male	1.10	(1.05–1.15)	1.56	(1.49–1.63)
Female	1.00		1.00	
Health insurance type				
National health insurance	1.00		1.00	
Medical aid	1.58	(1.40–1.79)	0.80	(0.70–0.91)
Income, N (%)				
Low (≤20th percentile)	1.27	(1.19–1.35)	1.25	(1.17–1.34)
Middle (21st–80th percentile)	0.95	(0.90–1.00)	1.16	(1.10–1.22)
High (≥81st percentile)	1.00		1.00	
Carstairs index, N (%)				
Disadvantaged neighborhood (below median)	1.03	(0.95–1.10)	1.08	(1.02–1.15)
Advantaged neighborhood (above median)	1.00		1.00	
Residential area				
Metropolitan	1.00		1.00	
Urban	1.11	(1.06–1.17)	1.04	(0.98–1.11)
Rural	1.47	(1.38–1.57)	1.14	(1.05–1.24)
Charlson comorbidity index^a^				
0–1	1.00		1.00	
2	1.46	(1.37–1.55)	1.25	(1.18–1.34)
3	2.15	(2.01–2.30)	1.67	(1.56–1.79)
≥4	4.41	(4.17–4.66)	2.69	(2.54–2.85)
Number of risk factors				
None	1.00		1.00	
with diabetes or dyslipidemia	0.57	(0.54–0.60)	0.63	(0.60–0.66)
with diabetes and dyslipidemia	0.26	(0.23–0.29)	0.36	(0.33–0.40)
Disability				
Normal	1.00		1.00	
Mild disability	1.47	(1.36–1.59)	1.11	(1.03–1.21)
Severe disability	3.00	(2.77–3.27)	1.44	(1.33–1.57)
Health screening during follow-up period				
1	1.00		1.00	
2	0.31	(0.29–0.34)	0.39	(0.36–0.42)
≥3	0.11	(0.10–0.12)	0.17	(0.16–0.19)

^a^calculated by extracting diabetes, hypertension, and hyperlipidemia from among comorbidity components

The HRs of individual household income for all-cause mortality in disadvantaged and advantaged neighborhoods are shown in Table 3. After stratifying the advantaged and disadvantaged neighborhoods according to the individual household income, the risk for all-cause mortality for patients who lived in a disadvantaged neighborhood was higher than for the individuals who lived in an advantaged neighborhood; this finding was applicable to the high income, middle income and low income groups, even though patients were in the same individual household income group. The adjusted HR of high income patients in an advantaged neighborhood and high income patients in a disadvantaged neighborhood was 1.10 (95% CI, 1.00–1.20; p-value = 0.05), while the adjusted HRs of middle income patients in advantaged neighborhood and disadvantaged neighborhood were 1.17 (95% CI, 1.08–1.26) and 1.27 (95% CI, 1.17–1.38), respectively. The adjusted HR for low income patients who lived in a disadvantaged neighborhood was higher than for those who lived in an advantaged neighborhood (HR, 1.35; 95% CI, 1.22–1.49 vs. HR, 1.28; 95% CI, 1.16–1.41).

**Table 3:** HRs of mortality according to individual household income in disadvantaged and advantaged neighborhoods

			Disadvantaged neighborhoods^c^				Advantaged neighborhoods^c^			
All-cause mortality			No. of deaths (deaths per 1,000 py^a^)		HR^b^	95% CI	No. of deaths (deaths per 1,000 py^a^)		HR^b^	95% CI
	Individual household income									
		High (≥81st percentile)	1,314	(38.6)	1.10	(1.00–1.20)^d^	1,127	(37.8)	1.00	
		Middle (21st–80th percentile)	2,168	(36.4)	1.27	(1.17–1.38)	1,607	(35.6)	1.17	(1.08–1.26)
		Low (≤20th percentile)	915	(47.7)	1.35	(1.22–1.49)	694	(50.7)	1.28	(1.16–1.41)

^a^py, person years

^b^adjusted hazard ratio after controlling for all covariates

^c^Disadvantaged and advantaged neighborhoods were distinguished on the basis of the mean for neighborhood Carstairs index, with disadvantaged neighborhoods having more-than-mean Carstairs index; a higher Carstairs index represents a more deprived neighborhood

^d^ *p*-value = 0.05

## Discussion

This study evaluated the association between individual income, neighborhood SES, and all-cause mortality in patients with hypertension. Our data showed that individual household income and neighborhood SES are important factors associated with the disparities in all-cause mortality in Korea. Specifically, lower household income and living in a disadvantaged neighborhood increased the risk for all-cause mortality. Moreover, the risk of mortality for all individual household income groups was increased for patients in the disadvantaged neighborhoods relative to the advantaged neighborhoods.

Our results are consistent with previous studies showing that patients in disadvantaged neighborhoods had a higher risk of mortality relative to their income equivalents living in advantaged neighborhoods, even though individuals had the same income level [15, 21, 25–27]. We posed the question, why do low income patients in disadvantaged neighborhoods have higher mortality? Yen and Kaplan [17] suggested “differential access to resources” as an explanation for these findings, and Hook [28] suggested lower “effective income” of low income patients living in advantaged neighborhoods as a reason for less “access to resources.” However, the health insurance programs in Korea provide universal coverage that has improved the accessibility to medical care. In addition, geographical accessibility to health care is better than many other countries because Korea is limited in size and has excellent transportation among its regions. The setting of our study is characterized by universal health care coverage and more equal access to primary education and other social services, suggesting that financial barriers are reduced and that access to resources is a less pronounced determinant of health. Therefore, why do socioeconomic disparities for mortality remain? One possible explanation is that more direct psychosocial factors, such as relative deprivation, hopelessness, lack of control, or loss of respect arising as a consequence of inequality affect individual health [29–31]. In addition, a lack of social cohesion or involvement, possibly linked to psychosocial issues, may contribute to the reduced health of low income patients in the advantaged areas.

Our findings demonstrated that individual household income represents different contextual effects on mortality. The causal linkages between the individual, neighborhood socioeconomic inequality, and poor health outcomes are not fully understood; several possible mechanisms could explain our findings. First, previous studies suggested “access to health care resources” as one possible explanation. However, even though the individual financial barriers for access to health care resources have been reduced, disparities may still exist in access to more expensive health care services, as well as in the number of physicians and the number of medical institutions between the regions. The second possibility is the role SES plays in how health is viewed, which may explain why the higher income individuals living in the more advantaged neighborhoods may be healthier. The ability of high income individuals to use their knowledge, money, power, prestige, and social connections would be reinforced by living in advantaged neighborhoods [32]. In addition, in comparison to low income individuals, high income individuals are quicker to adopt prevention strategies and take advantage of treatment innovations more rapidly [33]. Furthermore, in advantaged areas, health-related knowledge might be more readily shared and cultivated within the network of high income individuals [13]. In contrast, low income individuals are often more socially isolated, which decreases the likelihood of obtaining useful opinions or advice from others [34]. The third possibility is the lack of safe environments in disadvantaged neighborhoods, which reduces the possibility for exercise, thus potentiating an unhealthy lifestyle [35]. Moreover, socio-cultural norms regarding a healthy lifestyle could vary between advantaged and disadvantaged neighborhoods, which could impact the health of individuals and the risk for mortality. For example, studies have shown that environment-related risk factors, such as income, education, and unemployment are associated with mortality risk.

There were several limitations to our study. First, we included only high risk groups with hypertension. Our findings cannot be extrapolated to the general population without hypertension. Second, we could not consider factors such as lifestyle and education, which influence mortality risk, because these factors were not captured by the claims database. In addition, when we selected our study population with hypertension, we could not help using only ICD-10 code and oral antihypertensive medication, but use blood pressure. We did not have the ability to perform actual chart review, and as such, we acknowledge limitations associated with studies lacking detailed physiologic data such as blood pressure. Finally, we did not consider changes in the neighborhood deprivation status for the study participants who moved into a neighborhood with a different status during the study period.

Despite the limitations, our study had several strengths. First, to our knowledge, our study was the first to examine the relationship between individual income, neighborhood SES, and mortality in the context of universal health insurance. We used a prospective design and a relatively large sample, which yielded good statistical power, to detect the effects of neighborhood deprivation, and analyzed the data using a hierarchical frailty model. Second, we analyzed a representative sample of patients with hypertension using the nationwide representative cohort data. Third, we made an effort to increase the homogeneity of our study sample by restricting our study sample to patients who were newly diagnosed with hypertension.

## Conclusions

We found that combined effect between individual and neighborhood socioeconomic status on all-cause mortality in patients with newly diagnosed hypertension using a Cox proportional hazard frailty model. Our findings demonstrate how important it is for health professionals and policymakers to understand people within the context of their neighborhoods. The high mortality that we observed among patients of low household income who reside in high deprived neighborhoods suggested that they should focus on public health strategies for these people to reduce health inequalities.

## Additional file

Additional file 1: Table S1. The hypertensive status of subject according to individual household income. (DOCX 15 kb)

## Abbreviations

SES: Socioeconomic Status
KNHI: Korean National Health Insurance
CCI: Charlson Comorbidity Index
HR: Hazard Ratio
