# Supplementary material for: Disparities in socioeconomic status and neighborhood characteristics affect all-cause mortality in patients with newly diagnosed hypertension in Korea: a nationwide cohort study, 2002–2013
Source: Int J Equity Health. 2016 Jan 8;15:3. doi: 10.1186/s12939-015-0288-2 (PMC4705749; doi:10.1186/s12939-015-0288-2)
Supplement: Additional file 1: Table S1. — The hypertensive status of subject according to individual household income. (DOCX 15 kb) [file 12939_2015_288_MOESM1_ESM.docx]

|  |  | **Individual Household Income** | | | | | | Total (N=28,306) |
| --- | --- | --- | --- | --- | --- | --- | --- | --- |
|  |  | Low (N=4,801) | | Middle (N=14,541) | | High (N=8,964) | |  |
|  |  | N | (%) | N | (%) | N | (%) |  |
| **Hypertension** | |  |  |  |  |  |  |  |
|  | Primary hypertension (I10) | 4,166 | (16.7) | 12,839 | (51.6) | 7,897 | (31.7) | 24,902 |
|  | Hypertensive heart disease (I11) | 522 | (18.8) | 1,378 | (49.7) | 875 | (31.5) | 2,775 |
|  | Hypertensive renal disease (I12) | 51 | (19.6) | 123 | (47.3) | 86 | (33.1) | 260 |
|  | Hypertensive heart and renal disease (I13) | 62 | (16.8) | 201 | (54.5) | 106 | (28.7) | 369 |

Additional file 1: Table S1. The hypertensive status of subject according to individual household income
